# Feasibility study on full closed-loop control ventilation (IntelliVent-ASV^™^) in ICU patients with acute respiratory failure: a prospective observational comparative study

**DOI:** 10.1186/cc12890

**Published:** 2013-09-11

**Authors:** Jean-Michel Arnal, Aude Garnero, Dominik Novonti, Didier Demory, Laurent Ducros, Audrey Berric, Stéphane Yannis Donati, Gaëlle Corno, Samir Jaber, Jacques Durand-Gasselin

**Affiliations:** 1 Service de Réanimation Polyvalente, Hôpital Sainte Musse, 54 avenue Henri Sainte Claire Deville, 83056 Toulon, France; 2 Department of Medical Research, Hamilton Medical, 8 via Crusch, 7402 Bonaduz, Switzerland; 3 Hôpital Saint Eloi, CHU de Montpellier, 80 avenue Augustin Fliche, 34295 Montpellier, France

## Abstract

**Introduction:**

IntelliVent-ASV^™ ^is a full closed-loop ventilation mode that automatically adjusts ventilation and oxygenation parameters in both passive and active patients. This feasibility study compared oxygenation and ventilation settings automatically selected by IntelliVent-ASV^™ ^among three predefined lung conditions (normal lung, acute respiratory distress syndrome (ARDS) and chronic obstructive pulmonary disease (COPD)) in active and passive patients. The feasibility of IntelliVent-ASV^™ ^use was assessed based on the number of safety events, the need to switch to conventional mode for any medical reason, and sensor failure.

**Method:**

This prospective observational comparative study included 100 consecutive patients who were invasively ventilated for less than 24 hours at the time of inclusion with an expected duration of ventilation of more than 12 hours. Patients were ventilated using IntelliVent-ASV^™ ^from inclusion to extubation. Settings, automatically selected by the ventilator, delivered ventilation, respiratory mechanics, and gas exchanges were recorded once a day.

**Results:**

Regarding feasibility, all patients were ventilated using IntelliVent-ASV^™ ^(392 days in total). No safety issues occurred and there was never a need to switch to an alternative ventilation mode. The fully automated ventilation was used for 95% of the total ventilation time. IntelliVent-ASV^™ ^selected different settings according to lung condition in passive and active patients. In passive patients, tidal volume (V_T_), predicted body weight (PBW) was significantly different between normal lung (*n *= 45), ARDS (*n *= 16) and COPD patients (*n *= 19) (8.1 (7.3 to 8.9) mL/kg; 7.5 (6.9 to 7.9) mL/kg; 9.9 (8.3 to 11.1) mL/kg, respectively; *P *0.05). In passive ARDS patients, FiO_2 _and positive end-expiratory pressure (PEEP) were statistically higher than passive normal lung (35 (33 to 47)% versus 30 (30 to 31)% and 11 (8 to 13) cmH_2_O versus 5 (5 to 6) cmH_2_O, respectively; *P*< 0.05).

**Conclusions:**

IntelliVent-ASV^™ ^was safely used in unselected ventilated ICU patients with different lung conditions. Automatically selected oxygenation and ventilation settings were different according to the lung condition, especially in passive patients.

**Trial Registration:**

ClinicalTrials.gov: NCT01489085

## Introduction

Mechanical ventilation is widely used in intensive care units (ICU) to support patients' respiratory failure. Ventilatory support must be adapted to each patient's metabolism to provide the oxygen required in the blood (oxygenation function) and to eliminate carbon dioxide (CO_2_) (ventilation function). In conventional ventilation modes, physicians determine the oxygenation and ventilation settings manually. However, due to frequent changes in the physiological needs of ICU patients, these ventilation settings cannot be adjusted continuously, as this would require a continuous presence of caregivers at the bedside. To address this problem, ventilator management using an open-loop computerized decision support significantly reduces morbidity in patients with acute respiratory distress syndrome (ARDS) [1]. Closed-loop ventilation modes that automatically adjust some settings according to physiological input [2] represent a further advance. Closed-loop ventilation modes make it possible to select an individualized ventilation [3], to reduce workload [4], to improve patient-ventilator synchrony [5], and to reduce weaning duration in some settings [6-8].

IntelliVent-ASV^™ ^is a further development of adaptive support ventilation (ASV) that automatically adjusts oxygenation and ventilation settings in passive (absence of spontaneous breathing activity) and active patients. Ventilation settings (minute volume (MV), tidal volume (V_T_), and respiratory rate (RR)) are adjusted automatically to reach a target end-tidal CO_2 _(PETCO_2_) in passive patients and a target RR in active patients. Oxygenation settings (inspiratory fraction of oxygen (FiO_2_) and positive end-expiratory pressure (PEEP)) are adjusted automatically to reach a target pulse oxymetry (SpO_2_).

IntelliVent-ASV^™ ^has been studied during short periods of ventilation in passive and active ICU patients [9,10], after cardiac surgery [11], and in pediatric care [12]. In all situations, IntelliVent-ASV^™ ^was safe and delivered lower V_T_, peak inspiratory pressure (P_INSP_) and FiO_2 _in passive patients as compared to the controlled period in conventional ventilation. Up to now IntelliVent-ASV^™ ^has not been used for more than 24 hours in ventilated adult ICU patients. This prospective, observational feasibility study measured oxygenation and ventilation settings in ICU patients ventilated with IntelliVent-ASV^™ ^from inclusion to extubation or death. The primary objective was to compare ventilation delivered among three predefined lung conditions (normal lung, ARDS and chronic obstructive pulmonary disease (COPD)) in active and passive phases. The hypothesis was that IntelliVent-ASV^™ ^automatically selects different settings depending on the lung condition. The secondary objective was to assess the feasibility of IntelliVent-ASV^™ ^use defined as the number of safety events, the need to switch to conventional mode for any medical reason, or sensor failure.

## Patients and method

This prospective, observational, comparative study was conducted from November 2010 to September 2011 in the 12-bed medical-surgical adult ICU of Font Pré Hospital in Toulon (France). The institutional review board approved the protocol, which was also declared at the Commission Nationale Informatique et Liberté (CNIL), and informed consent was obtained from each patient's next of kin.

### Patients

Consecutive patients admitted in the ICU between November 2010 and September 2011 were included if they met the inclusion criteria and none of the exclusion criteria (Table 1). Patients were mechanically ventilated using a Hamilton-S1 ventilator (Hamilton Medical AG, Rhäzüns, Switzerland) with IntelliVent-ASV^™ ^software (v1.10) using one SpO_2 _sensor. A heated humidifier was used for gas conditioning.

**Table 1:** Inclusion and noninclusion criteria.

Inclusion criteria	Exclusion criteria
1. Patient invasively ventilated for less than 24 hours 2. Expected duration of ventilation more than 12 hours 3. Ventilator S1 available (4 out of 12 beds)	1. Readiness to wean criteria fulfilled 2. Bronchopleural fistula 3. Brain death

### IntelliVent-ASV^™^

#### Basic principles

IntelliVent-ASV^™ ^is a mode in which the physician selects the oxygenation as a target SpO_2 _and ventilation as a target PETCO_2 _individually for each patient. IntelliVent-ASV^™ ^combines a ventilation controller that adjust P_INSP _and RR and an oxygenation controller to adjust FiO_2 _and PEEP. The ventilator delivers a volume-targeted pressure-controlled breath equivalent to an adaptive pressure control in passive patients, and an adaptive pressure support in active patients.

At initiation, physicians have to set predicted body weight (PBW) [13] and the clinical condition (normal lung, ARDS, chronic hypercapnia, or brain injury). For each clinical condition, default target ranges of PETCO_2 _and SpO_2 _are defined, which can be manually adjusted by the user. When chronic hypercapnia and brain injury are selected, PEEP must be manually set and the oxygenation controller selects FiO_2 _automatically.

#### Safety features

Safety limits were designed in the protocol. The ventilation controller was deactivated in passive patients if plateau pressure (P_PLAT_) increased above 35 cmH_2_O, or V_T_/PBW above 10 mL/kg (12 mL/Kg for COPD), or RR above 35 breath/min for more than 30 seconds [9], or in the case of severe respiratory acidosis with pH below 7.20 [14]. In active patients, the ventilation controller was deactivated if RR was above 40 breaths/min for more than 30 seconds or in case of patient's severe agitation. The oxygenation controller was deactivated if SpO_2 _was below 85% for more than 1 minute.

#### Settings, adjustment and weaning

IntelliVent-ASV^™ ^was used from inclusion to extubation or death. At initiation, physicians set the patient's gender and height and clinical condition (ARDS, chronic hypercapnia, or brain injury).

Clinical condition, PETCO_2 _and SpO_2 _targets, inspiratory and expiratory triggers, rise time and alarm settings were reassessed at least twice daily during the morning and evening rounds. When arterial blood gas was measured, the end-tidal to arterial PCO_2 _gradient was calculated according to: PaCO_2 _to PETCO_2_. Because the ventilation controller uses PETCO_2 _to adjust MV, target PETCO_2 _ranges were adjusted in passive patients when the end-tidal to arterial PCO_2 _gradient was above 5 mmHg. This adjustment was mostly required for patients with severe ventilation perfusion

Sedation was performed according to the unit protocol. The standard unit weaning protocol was applied, based on a daily screening of readiness to wean criteria and a weaning trial (equivalent of a T-tube trial) managed by the nurse in charge. Weaning trial was performed using PEEP of 5 cmH_2_O and target MV set at 25 mL/kg PBW/min, which through experience our team found to be equivalent of pressure support (PS) of 5 to 7 cmH_2_O. The FiO_2 _controller was still active during the weaning trial, which lasted 30 minutes. At the end of the weaning trial, the physician in charge decided to extubate the patient or to return to previous ventilation. Patients at risk of respiratory distress after extubation received noninvasive ventilation in sequential sessions for 24 hours [15].

### Data collection

At inclusion patients were classified into one of the four lung conditions (normal lung, acute lung injury (ALI)/ARDS, COPD, or others) according to their medical history, chest examination, chest radiography, arterial blood gas analysis, and any other examination result that may have been performed. This classification was independent from the clinical condition selected on the ventilator. Normal lung was selected for patients with no underlying respiratory disease, normal chest radiography, and arterial pressure of oxygen (PaO_2_)/FiO_2 _ratio of 300 mmHg or higher; ALI/ARDS as defined by the American-European Consensus Conference [16]; COPD as defined by the 'GOLD' criteria [17]; others combines chest wall stiffness patients (presence of kyphoscoliosis, morbid obesity with a body mass index over 35 kg/m^2^, or a neuromuscular disorder) and acute respiratory failure (PaO_2_/FiO_2 _ratio of 300 mmHg or less without the ALI/ARDS criteria from the American-European Consensus Conference [16]).

For any given patient each ventilation day was categorized as passive or active, the latter being defined by the patient's spontaneous RR over 75% of the total RR. Settings automatically selected, manual settings, ventilation delivered, respiratory mechanics, and physiologic variables were collected once a day at 7 am using the ventilator display. Time of data collection was chosen to be apart from nursing care and medical procedures. Controller deactivation was defined as the need to stop the automated function and manually adjust the settings. All episodes of controller deactivation were recorded.

### Statistical methods

Values are expressed as medians (25^th ^to 75^th ^interquartile range). Kruskal-Wallis analysis of variance (ANOVA) was used to compare values between each type of lung conditions for active and passive breathing patients. Statistical significance was assumed for *P *value no greater than 0.05. Statistical analysis was performed using SigmaStat software (version 3.5, Systat Software, Inc., Chicago, IL, USA).

## Results

**Figure 1:**
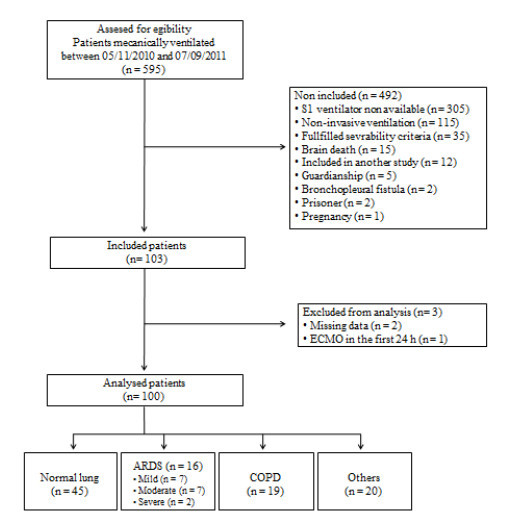
**Study flow diagram**.

During the study period, 789 patients were admitted in the ICU of which 103 patients were included in the study. Three patients were not analyzed (two for missing data and one was transferred for extracorporeal membrane oxygenation (ECMO) rapidly after inclusion). Thus, 100 patients were analyzed (Figure 1). Table 2 presents patient characteristics at inclusion, lung condition and outcomes. Seventy-seven patients were ventilated for longer than 24 hours.

**Table 2:** Baseline characteristics of the study population, lung condition at inclusion, and outcomes.

Number of patients	100
Age (years)	73 (64-79)

Gender (M/F)	58/42

Actual body weight (kg)	85 (60-80)

Predicted body weight (kg)	60 (53-68)

SAPS II	56 (48-69)

Mechanical ventilation duration before inclusion (days)	0.5 (0.0-0.8)

Sedation at inclusion (n)	73

Vasopressors at inclusion (n)	52

Passive ventilation duration (days)	1.0 (0.0-2.5)

Active mechanical ventilation duration (days)	1.2 (0.5-3.0)

Duration of invasive mechanical ventilation (days)	3.0 (2.0-7.0)

Post-extubation noninvasive mechanical ventilation (n)	26

Total duration of mechanical ventilation (days)	4.0 (2.0-7.0)

Reintubation (n)	2

Duration of ICU stay (days)	5 (2-10)

Duration of hospital stay (days)	11 (4-22)

ICU mortality (n)	31

Hospital mortality (n)	41

Data are presented as median (25^th ^to 75^th ^quartile). SAPS, simplified acute physiology score.

**Figure 2:**
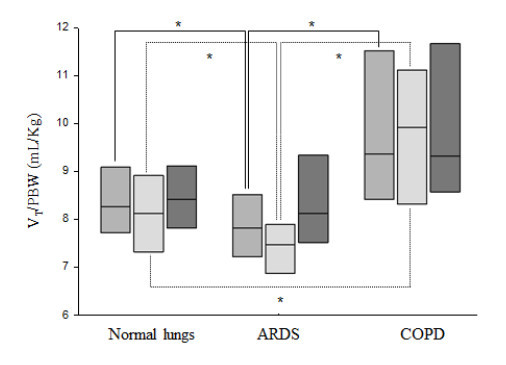
**Tidal volume selected by IntelliVent-ASV^™^: Tidal volume on predicted body weight ratio for normal lung patients, ARDS and COPD patients**. For each lung condition, all patients, passive patients and active patients are shown on the left, middle and right box plot, respectively. Comparisons used a Kruskal-Wallis analysis of variance with a Dunn's post hoc test. *P ≤0.05. ARDS, acute respiratory distress syndrome; COPD, chronic obstructive pulmonary disease.

**Figure 3:**
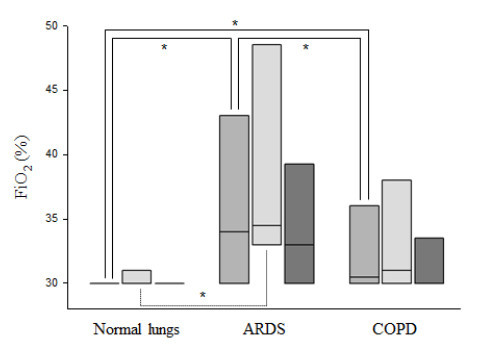
**FiO_2 _selected by IntelliVent-ASV^™^: FiO_2 _for normal lung patients, ARDS and COPD patients**. For each lung condition, all patients, passive patients and active patients are shown on the left, middle and right box plot, respectively. Comparisons used a Kruskal-Wallis analysis of variance with a Dunn's post hoc test. *P ≤0.05. ARDS, acute respiratory distress syndrome; COPD, chronic obstructive pulmonary disease; FiO_2_, inspiratory fraction of oxygen (%).

**Figure 4:**
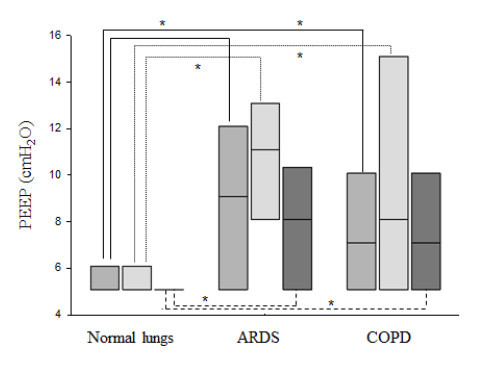
**PEEP selected by IntelliVent-ASV^™^: PEEP for normal lung patients, ARDS and COPD patients**. For each lung condition, all patients, passive patients and active patients are shown on the left, middle and right box plot, respectively. Comparisons used a Kruskal-Wallis analysis of variance with a Dunn's post hoc test. *P ≤0.05. ARDS, acute respiratory distress syndrome; COPD, chronic obstructive pulmonary disease; PEEP, positive end-expiratory pressure (cmH_2_O).

In passive and active ventilation-days, MV, V_T_/PBW, PEEP, FiO_2_, P_INSP_, and RR were statistically different based on lung condition (Table 3, Figure 2, 3, 4). In passive ventilation days, V_T_/PBW was significantly different between normal lung, ARDS and COPD patients (8.1 (7.3 to 8.9) mL/kg; 7.5 (6.9 to 7.9) mL/kg; 9.9 (8.3 to 11.1) mL/kg, respectively; *P *<0.05) (Table 4 and Figure 2). In passive ARDS ventilation days, FiO_2 _and PEEP were statistically higher than in passive normal lung patients (35 (33 to 47)% versus 30 (30 to 31)% and 11 (8 to 13) cmH_2_O versus 5 (5 to 6) cmH_2_O, respectively; *P *< 0.05) (Table 4 and Figure 3 and 4). In active ventilation days, V_T_/PBW was significantly higher in COPD patients as compared to normal lung and ARDS patients (9.3 (8.6 to 11.6) mL/kg; 8.4 (7.8 to 9.1) mL/kg; 8.1 (7.5 to 9.3) mL/kg, respectively; *P *<0.05) (Table 5 and Figure 2). In active ARDS and COPD ventilation days, PEEP was significantly higher than in normal lung patients (8 (5 to 10) cmH_2_O, 7 (5 to 10) cmH_2_O, and 5 (5 to 5) cm H_2_O, respectively; *P *<0.05) (Table 5 and Figure 4).

**Table 3:** Settings, delivered ventilation, respiratory mechanics and blood gas results according to the lung condition in all patients.

	1 Normal lung	2 ARDS	3 COPD	4 Others	***P (ANOVA)***	Post hoc comparison (*P ≤0.05*)
Number of patients	45	16	19	20		

Number of days	139	90	79	84		

MV (L/min)	9.3 (7.5-11.7)	9.7 (7.8-13.3)	9.4 (6.4-11.9)	7.5 (6.6-10.8)	*0.001*	1 vs. 4

V_T_/PBW (mL/kg)	8.3 (7.7-9.1)	7.8 (7.2-8.5)	9.4 (8.4-11.5)	8.0 (7.3-9.2)	*<0.001*	1 vs. 3; 2 vs. 3; 3 vs. 4

PEEP (cmH_2_O)	5 (5-6)	9 (5-12)	7 (5-10)	8 (5-11)	*<0.001*	1 vs. 2; 1 vs. 3; 1 vs. 4

FiO_2 _(%)	30 (30-30)	34 (30-43)	31 (30-36)	30 (30-41)	*<0.001*	1 vs. 2; 1 vs. 3; 1 vs. 4; 2 vs. 3

P_INSP _(cmH_2_O)	21 (18-25)	25 (20-30)	24 (19-38)	26 (22-30)	*<0.001*	1 vs. 2; 1 vs. 3; 1 vs. 4

RR (breath/min)	19 (16-23)	20 (16-26)	15 (12-19)	18 (14-22)	*<0.001*	1 vs. 3; 2 vs. 3; 2 vs. 4

RC_EXP _(s)	0.61 (0.50-0.75)	0.50 (0.43-0.58)	0.91 (0.64-1.22)	0.56 (0.46-0.66)	*<0.001*	1 vs. 2; 1 vs. 3; 2 vs. 3; 3 vs. 4

PaO_2_/FiO_2 _(mm Hg)	326 (267-380)	206 (172-252)	260 (206-328)	241 (189-304)	*<0.001*	1 vs. 2; 1 vs. 3; 1 vs. 4; 2 vs. 3; 2 vs. 4

PaO_2 _(mm Hg)	100 (85-117)	76 (69-84)	86 (74-105)	82 (73-95)	*<0.001*	1 vs. 2; 1 vs. 3; 1 vs. 4; 2 vs. 3; 2 vs. 4

pH	7.36 (7.30-7.41)	7.35 (7.23-7.42)	7.30 (7.25-7.35)	7.33 (7.26-7.41)	*<0.001*	1 vs. 3; 2 vs. 3

PaCO_2 _(mm Hg)	35 (31-40)	41 (35-49)	45 (37-53)	39 (34-46)	*<0.001*	1 vs. 2; 1 vs. 3; 1 vs. 4

Values represent the median of all data. Comparisons used a Kruskal-Wallis analysis of variance (ANOVA) with a Dunn's post hoc test. MV, minute volume, V_T_/PBW, tidal volume on predicted body weight ratio, PEEP, positive end-expiratory pressure, P_INSP_, peak inspiratory pressure, RR, respiratory rate, RC_EXP_, expiratory time constant, vs., versus.

**Table 4:** Settings, delivered ventilation, respiratory mechanics and blood gas results according to the lung condition in passive patients

	1 Normal lung	2 ARDS	3 COPD	4 Others	***P (ANOVA)***	Post hoc comparison (*P ≤ 0.05*)
Number of patients	27	13	12	15		

Number of days	63	36	23	40		

MV (L/min)	8.4 (6.73-9.7)	7.9 (6.1-8.8)	7.8 (5.3-9.0)	7.2 (5.3-8.5)	*0.076*	

V_T_/PBW (mL/kg)	8.1 (7.3-8.9)	7.5 (6.9-7.9)	9.9 (8.3-11.1)	8.0 (7.5-9.2)	*<0.001*	1 vs. 2; 1 vs. 3; 2 vs. 3; 2 vs. 4

PEEP (cmH_2_O)	5 (5-6)	11 (8-13)	8 (5-15)	10 (5-13)	*<0.001*	1 vs. 2; 1 vs. 3; 1 vs. 4

FiO_2 _(%)	30 (30-31)	35 (33-47)	31 (30-38)	31 (30-53)	*<0.001*	1 vs. 2; 1 vs. 4

P_INSP _(cmH_2_O)	21 (19-23)	27 (24-30)	27 (23-39)	26 (23-30)	*<0.001*	1 vs. 2; 1 vs. 3; 1 vs. 4

RR (breath/min)	17 (15-21)	16 (15-20)	13 (12-15)	15 (13-18)	*<0.001*	1 vs. 3; 1 vs. 4; 2 vs. 3

RC_EXP _(s)	0.58 (0.50-0.72)	0.47 (0.40-0.54)	1.22 (0.68-1.37)	0.57 (0.46-0.72)	*<0.001*	1 vs. 2; 1 vs. 3; 2 vs. 3; 2 vs. 4; 3 vs. 4

R_INSP _(cm H_2_O/s/L)	15 (12-17)	11 (10-16)	21 (17-26)	16 (12-20)	*<0.001*	1 vs. 3; 2 vs. 3; 2 vs. 4

C_STAT _(L/cm H_2_O)	39 (35-54)	29 (26-37)	53 (46-61)	32 (28-40)	*<0.001*	1 vs. 2; 1 vs. 4; 2 vs. 3; 3 vs. 4

I/E ratio	0.61 (0.43-0.77)	0.91 (0.83-1.00)	0.43 (0.32-0.50)	0.77 (0.61-1.00)	*<0.001*	1 vs. 2; 2 vs. 3; 3 vs. 4

P_PLAT _(cm H_2_O)	18 (17-20)	26 (23-29)	23 (17-31)	24 (21-28)	*<0.001*	1 vs. 2; 1 vs. 3; 1 vs. 4

PaO_2_/FiO_2 _(mm Hg)	328 (264-366)	201 (155-256)	253 (206-295)	208 (147-301)	*<0.001*	1 vs. 2; 1 vs. 3; 1 vs. 4

PaO_2 _(mm Hg)	99 (85-114)	74 (66-88)	83 (77-94)	82 (70-95)	*<0.001*	1 vs. 2; 1 vs. 4

pH	7.33 (7.27-7.40)	7.24 (7.15-7.34)	7.29 (7.20-7.31)	7.29 (7.22-7.34)	*0.002*	1 vs. 2; 1 vs. 3

PaCO_2 _(mm Hg)	38 (35-42)	49 (45-57)	49 (44-67)	43 (38-51)	*<0.001*	1 vs. 2; 1 vs. 3; 1 vs. 4; 2 vs. 4; 3 vs. 4

Values represent the median of all data. Comparisons used a Kruskal-Wallis analysis of variance (ANOVA) with a Dunn's post hoc test. MV, minute volume, V_T_/PBW, tidal volume on predicted body weight ratio, PEEP, positive end-expiratory pressure, P_INSP_, peak inspiratory pressure, RR, respiratory rate, RC_EXP_, expiratory time constant, R_INSP_, inspiratory resistances, C_STAT_, static compliance, I/E, inspiratory on expiratory time ratio, P_PLAT_, plateau pressure, vs., versus.

**Table 5:** Settings, delivered ventilation, respiratory mechanics and blood gas results according to the lung condition in active patients.

	1 Normal lung	2 ARDS	3 COPD	4 Others	*P (ANOVA)*	Post hoc comparison (*P ≤0.05*)
Number of patients	33	13	18	15		

Number of days	76	54	57	44		

MV (L/min)	10.1 (8.0-13.0)	12.5 (11.0-14.9)	11.1 (8.3-13.9)	9.2 (7.2-12.0)	*<0.001*	1 vs. 2; 2 vs.

V_T_/PBW (mL/kg)	8.4 (7.8-9.1)	8.1 (7.5-9.3)	9.3 (8.6-11.6)	7.7 (7.2-9.3)	*<0.001*	1 vs. 3; 2 vs. 3; 3 vs. 4

PEEP (cmH_2_O)	5 (5-5)	8 (5-10)	7 (5-10)	7 (5-10)	*<0.001*	1 vs. 2; 1 vs. 3; 1 vs. 4

FiO_2 _(%)	30 (30-30)	33 (30-38)	30 (30-33)	30 (30-34)	*<0.001*	1 vs. 2

P_INSP _(cmH_2_O)	21 (16-26)	24 (16-28)	22 (17-36)	24 (21-28)	*0.052*	

RR (breath/min)	21 (18-26)	25 (20-29)	18 (14-20)	21 (17-26)	*<0.001*	1 vs. 3; 2 vs. 3; 3 vs. 4

RC_EXP _(s)	0.66 (0.51-0.78)	0.52 (0.45-0.61)	0.86 (0.64-1.12)	0.53 (0.46-0.65)	*<0.001*	1 vs. 2; 1 vs. 3; 2 vs. 3; 3 vs. 4

PaO_2_/FiO_2 _(mm Hg)	322 (278-387)	213 (173-250)	273 (206-341)	253 (222-311)	*<0.001*	1 vs. 2; 1 vs. 3; 1 vs. 4; 2 vs. 3; 2 vs. 4

PaO_2 _(mm Hg)	102 (85-121)	77 (70-84)	89 (72-107)	83 (75-94)	*<0.001*	1 vs. 2; 1 vs. 3; 1 vs. 4; 2 vs. 3

pH	7.38 (7.34-7.44)	7.40 (7.35-7.43)	7.33 (7.25-7.37)	7.37 (7.30-7.42)	*<0.001*	1 vs. 3; 2 vs. 3; 3 vs. 4

PaCO_2 _(mm Hg)	32 (28-37)	37 (30-40)	41 (34-50)	36 (32-44)	*<0.001*	1 vs. 3; 1 vs. 4

Values represent the median of all data. Comparisons used a Kruskal-Wallis analysis of variance (ANOVA) with a Dunn's post hoc test. MV, minute volume, V_T_/PBW, tidal volume on predicted body weight ratio, PEEP, positive end-expiratory pressure, P_INSP_, peak inspiratory pressure, RR, respiratory rate, RC_EXP_, expiratory time constant, vs., versus.

Regarding feasibility, all patients were ventilated using IntelliVent-ASV^™ ^from inclusion to extubation or death (392 days in total) and no safety issues occurred. There was never a medical need to switch to another ventilation mode. The fully automated ventilation was used for 95% of total ventilation time, and partial automated ventilation for 5% of ventilation time (ventilation controller alone: 4%; oxygenation controller alone: 1%). The ventilation controller was deactivated in two patients for one day because of an increased CO_2_) gradient resulting in severe respiratory acidosis. PEEP and FiO_2 _controllers were deactivated for one day in seven patients because of a poor SpO_2 _quality measurement (five patients in shock, one patient with therapeutic hypothermia and one patient with severe chronic arterial disease). PEEP controller was deactivated in three patients; one COPD for manual adjustment according to intrinsic PEEP, one after a pneumothorax resulting from subclavicular catheter insertion and one ARDS patient for manual adjustment according to transpulmonary pressure. The FiO_2 _controller was deactivated in one COPD patient because of hyperoxia.

## Discussion

This observational study measured ventilation and oxygenation parameters automatically determined by IntelliVent-ASV^™ ^in 100 unselected ventilated ICU patients. IntelliVent-ASV^™ ^automatically selected different ventilation and oxygenation parameters according to lung condition especially for passive breathing patients. It was feasible to use IntelliVent-ASV^™ ^with no resulting safety issues for patients and with very few sensor failures.

In passive patients, ventilation and oxygenation parameters determined by IntelliVent-ASV^™ ^were different according to lung condition. In normal lung patients, V_T_/PBW was 8.1 (7.3 to 8.9) mL/kg with P_PLAT _at 18 (17 to 20) cmH_2_O and a PEEP at 5 (5 to 6) cmH_2_O. Current recommendations regarding normal lung patients are to set V_T_/PBW between 6 and 8 mL/kg in patients at risk of ARDS, and ≤10 mL/kg in patients without risk factors and to set PEEP between 5 and 12 cmH_2_O [18,19]. Thus, ventilation selected by IntelliVent-ASV^™ ^in patients with normal lungs at the onset of mechanical ventilation is in line with current recommendations to prevent ventilator-induced lung injuries (VILI). FiO_2 _selected in passive normal lung patients was 30 (30 to 31)%. A recent retrospective database study found that hyperoxia is commonly seen in the ICU and in most cases does not lead to the adjustment of ventilator settings if FiO_2 _is below or equal to 40% [20]. There have been studies in humans that report the physiological effects of hyperoxia including impaired myocardial blow flow [21], increased myocardial consumption [22], and reduced cerebral blood flow due to arterial vasoconstriction [23]. Thus, in normal lung patients, where hyperoxia is easily obtained, automatic adjustment of FiO_2 _should minimize FiO_2 _levels, prevent unnecessary hyperoxia and avoid potential systemic oxygen toxicity.

In passive ARDS patients, V_T_/PBW was 7.5 (6.1 to 8.8) mL/kg with P_PLAT _at 26 (23 to 29) cmH_2_O and a PEEP at 11 (8 to 13) cmH_2_O. These results are in line with current recommendations to use a protective ventilation strategy in order to prevent VILI. A meta-analysis of protective ventilation trials in ARDS patients found a favorable effect for V_T_/PBW less than 7.7 mL/kg [24]. Even though P_PLAT _and V_T_/PBW are not good surrogates to assess lung stress and strain [25], these values are easily measured at the bedside and are widely used. A large international observational study showed that the current mechanical ventilation practice in ARDS patients is to set a median V_T_/PBW between 6 and 8 mL/kg with a median PEEP at 5 to 12 cmH_2_O [26]. A large multicenter observational study in Spain observed 255 ARDS patients and found a V_T_/PBW at 7.2 ± 1.1 mL/kg, P_PLAT _at 26 ± 5 cmH_2_O, and PEEP at 9.3 ± 2.4 cmH_2_O [27]. Thus, IntelliVent-ASV^™ ^selects V_T_/PBW and PEEP that are in line with current recommendations and current practices. Despite the evidence showing that a reduced V_T _strategy is associated with improved outcomes, physicians still routinely use higher V_T _than recommended [28-30]. The main reasons for physicians' underuse of low V_T _ventilation are the use of actual body weight instead of PBW in the calculation of V_T_, a general underrecognition of ARDS in clinical practice [31], physician concern about increasing RR with a potential risk of dynamic hyperinflation [32], and concern about permissive hypercapnia and acidosis [33]. IntelliVent-ASV^™ ^automatically computes the PBW based on height and gender, automatically recognizes short expiratory time constant (RC_EXP_) and adjusts V_T _and RR accordingly, prevents dynamic hyperinflation in passive patients by adjusting expiratory time according to RC_EXP_, and allows a moderate permissive hypercapnia when P_INSP _is above 25 cmH_2_O. Overall, IntelliVent-ASV^™ ^is a useful way to help implement protective ventilation.

In ARDS patients, IntelliVent-ASV^™ ^selected oxygenation settings that combined a moderately high PEEP (11 (8 to 13) cmH_2_O) with a low FiO_2 _(35 (33 to 47)%). This combination follows the higher PEEP-FiO_2 _table [34], which complies with the open lung concept [35] in order to prevent atelectrauma.

In passive COPD patients, V_T_/PBW was 9.9 (8.3 to 11.1) mL/kg. There is no consensus as to the optimal V_T _in passive COPD [36,37]. However, because end-expiratory lung volume is increased in COPD, the lung strain associated with relatively high V_T _remains limited [25].

In active patients, the difference in V_T_/PBW between each lung condition was less than in passive patients. This is explained by the range of RC_EXP_, which was narrower in the lung conditions in active compared to passive patients. As a consequence, there is not a significant difference between the target V_T_/PBW determined by the ASV algorithm for the different lung conditions. In addition, V_T_/PBW in active patients is very dependent on patient's drive, which may be increased as a result of elements such as metabolic acidosis, pain, discomfort, anxiety, and the sedation used. It should be noted that active ARDS patients are in the weaning phase after severe hypoxemia has been resolved.

In this study, assessment of feasibility was defined by the number of safety events, the need to switch to another mode and sensor failure. As in previous studies [9-11], there was no safety issue according to the predefined safety criteria. None of the users were uncomfortable with IntelliVent-ASV^™ ^to the extent that it was necessary to switch to conventional ventilation. Overall, physicians had to deactivate one controller for only 5% of the total ventilation time. The main reasons were a large CO_2 _gradient and SpO_2 _signal of poor quality [38,39]. Considering the large numbers of ventilation days and the relatively small number of problems, this study shows that the use of IntelliVent-ASV^™ ^is feasible on a daily basis in unselected ICU patients with different lung conditions. However, monitoring of patients is still required, especially for the CO_2 _gradient in patients with large ventilation/perfusion ratio disturbances. Also, in case of sudden decrease of PETCO_2 _due to a shock or pulmonary embolism, there is a risk of hypoventilation. The current software does not sound an alarm when PETCO_2 _and MV change but does sound an alarm when the low PETCO_2 _or MV threshold is reached.

The main limitation of this study is that it is observational, which does not allow comparison with conventional modes in terms of settings or outcome. In addition, numbers of severe ARDS patients were too small to draw definitive conclusions on safety. Studies in this subgroup population are required. Data were collected once a day and not continuously. Results would possibly be different if measured continuously or with a higher sampling rate. However, the times that were selected to collect the data were chosen specifically to be isolated from nursing care and medical procedures. Adaptation of IntelliVent-ASV^™ ^during nursing care and medical procedures deserves additional studies. Finally, this study was performed in a unit with a large experience of ASV use. Applicability of results may be different in other settings.

## Conclusions

This observational study found that IntelliVent-ASV^™ ^seems safe for all ICU duration in ventilated unselected ICU patients with different lung conditions. More data is required for specific populations, in particular severe ARDS patients. Automatically selected oxygenation and ventilation settings were different according to the lung condition, especially in passive patients, and are in line with current recommendations. These results are a step forward in the implementation of closed-loop mode in daily practice in the ICU.

## Key messages

• Use of a full closed-loop ventilation mode seems safe in nonselected ICU patients.

• The full closed-loop ventilation mode can be used during 95% of the total ventilation time.

• IntelliVent-ASV™ selects different ventilation and oxygenation settings in passive and active patients with normal lungs, ARDS, and COPD.

## Abbreviations

ALI: acute lung injury; ANOVA: analysis of variance; ARDS: acute respiratory distress syndrome; ASV: adaptive support ventilation; CNIL: Commission Nationale Informatique et Liberté; CO_2: _carbon dioxide (mmHg); COPD: chronic obstructive pulmonary disease; C_STAT: _static compliance (mL/cmH_2_O); ECMO: extracorporeal membrane oxygenation; PETCO_2: _end-tidal CO2 (mmHg); FiO_2: _inspiratory fraction of oxygen (%); ICU: intensive care unit; I/E: inspiratory on expiratory time ratio; MV: minute volume; PaCO_2: _arterial pressure of CO_2 _(mm Hg): PaO_2: _arterial pressure of oxygen (mmHg): PBW: predicted body weight (kg); PEEP: positive end-expiratory pressure (cmH_2_O); PEEP_TOT: _total positive end-expiratory pressure (cmH_2_O); P_INSP_: peak inspiratory pressure (cmH_2_O); P_PLAT: _plateau pressure (cmH_2_O); PS: pressure support; RC_EXP: _expiratory time constant (s); R_INSP: _inspiratory resistances (cmH_2_O.s/L); RR: respiratory rate (breath/min); SAPS: simplified acute physiology score; SpO_2: _pulse oxymetry (%); V_T: _tidal volume (mL); VILI: ventilator-induced lung injuries.

## Competing interests

This study was promoted by the Centre Hospitalier Intercommunal de Toulon La Seyne sur Mer. The hospital research unit received an 8000 € grant from Hamilton Medical to cover the submission fees and insurance. At the time of the study, JMA was working as a full-time physician in the ICU. He was supported by Hamilton Medical in presenting the results at international conferences. JMA has been working part-time for Hamilton Medical as a medical research manager since July 2012. DN is the head of research in Hamilton Medical.

AG, DD, LD, AB, SYD, GC, SJ, and JDG have no conflict of interest.

## Author's contributions

JMA designed the study, wrote the protocol, collected data, analyzed the results and drafted the manuscript. AG has made a substantial contribution to writing the protocol, collecting data, analyzing the results and drafting the manuscript. DD, LD, AB, SYD, and GC have made substantial contributions to collecting the data and revising the manuscript. DN, SJ and JDG have made substantial contributions to interpreting the results and revising the manuscript. All authors read and approved the final manuscript.
